# Safety and efficacy of lenvatinib combined with camrelizumab plus transcatheter arterial chemoembolization for unresectable hepatocellular carcinoma: A two-center retrospective study

**DOI:** 10.3389/fonc.2022.982948

**Published:** 2022-09-12

**Authors:** Bo Sun, Lijie Zhang, Tao Sun, Yanqiao Ren, Yanyan Cao, Weihua Zhang, Licheng Zhu, Yusheng Guo, Yuxi Gui, Fengyong Liu, Lei Chen, Fu Xiong, Chuansheng Zheng

**Affiliations:** ^1^ Department of Radiology, Union Hospital, Tongji Medical College, Huazhong University of Science and Technology, Wuhan, China; ^2^ Key Laboratory of Molecular Imaging of Hubei Province, Wuhan, China; ^3^ Department of Interventional Radiology, Union Hospital, Tongji Medical College, Huazhong University of Science and Technology, Wuhan, China; ^4^ Department of Interventional Radiology, The Fifth Medical Center of Chinese, People’s Liberation Army (PLA) General Hospital, Beijing, China

**Keywords:** lenvatinib, transcatheter arterial chemoembolization, camrelizumab, hepatocellular carcinoma, efficacy, safety

## Abstract

**Objectives:**

To compare the safety and efficacy of lenvatinib (LEN) combined with camrelizumab plus transcatheter arterial chemoembolization (TACE-LEN-C) and TACE combined with LEN (TACE-LEN) in patients with unresectable hepatocellular carcinoma (uHCC).

**Methods:**

Eighty-three patients with uHCC treated with TACE-LEN-C or TACE-LEN from September 2018 to May 2021 were enrolled in this retrospective study. Overall survival (OS), progression-free survival (PFS), local tumor response, and adverse events (AEs) were evaluated. Univariate and multivariate analyses were used to determine the factors affecting survival.

**Results:**

There were 31 patients in the TACE-LEN-C group and 52 patients in the TACE-LEN group. The median follow-up period was 14.2 months (range 7.2–25.2 months) in the whole study. The combination of triple therapy was found to significantly prolong the PFS (12.5 months vs. 6.6 months, *P*<0.001) and OS (18.9 months vs. 13.9 months, *P*<0.001. In terms of tumor response, the combination demonstrated a higher objective response rate (71% vs. 42.3% by the modified Response Evaluation Criteria in Solid Tumors, *P*=0.023) without a statistically significant difference in the disease control rate (93.5% in TACE-LEN-C, 80.8% in TACE-LEN, *P*=0.195). In the multivariate analysis, two independent factors affecting PFS were identified: number of tumors and treatment. Three independent factors affected OS: number of tumors, Barcelona Clinic Liver Cancer (BCLC) stage, and treatment. All the AEs were tolerable.

**Conclusion:**

TACE-LEN-C is a safe and effective treatment for patients with uHCC, and could be a potential treatment option.

## Introduction

Hepatocellular carcinoma (HCC) accounts for nearly 85% of all liver cancer patients, and is the third leading cause of cancer-related death. (1) The prognosis of HCC remains poor, with the maximum 5-year survival estimated at 18%. (2) Surgery and radiofrequency ablation are the curative treatments for HCC patients; (3) however, approximately 70% of the HCC cases are unresectable at diagnosis. (4) The median survival of patients with unresectable HCC (uHCC) is 16 months. (5) Systemic treatment is the first-line treatment recommended for patients with advanced HCC, including sorafenib, Lenvatinib, and atezolizumab + bevacizumab. (6)

Transcatheter arterial chemoembolization (TACE) is the standard treatment for intermediate HCC recommended by most clinical practice guidelines. (7–9) TACE can cause tumor regression in up to 50% of the patients with HCC, resulting in survival benefits. (10) However, as a palliative approach, TACE has not been universally successful in controlling liver cancer growth because of the high rate of incomplete embolization and changes in the tumor microenvironment (TME) after embolization. (11) Thus, many combination strategies have been explored for the treatment of unresectable HCC to improve the long-term outcomes of HCC patients treated with TACE. Molecularly targeted drugs are common options for combination with locoregional therapy. (12–16) However, the results did not demonstrate the expected synergistic results of combining TACE with molecular targeted drugs versus TACE alone for uHCC. Although the TACTICS study demonstrated that concurrent sorafenib therapy might delay tumor progression following TACE, the latest results showed no survival benefits compared with TACE alone. (17, 18)

Tumor microenvironment in HCC is strongly immunosuppressive, expressing a high level of immune checkpoint inhibitors(ICIs), such as programmed death-1 (PD-1), cytotoxic T-lymphocyte antigen 4 (CTLA-4), lymphocyte activating gene 3 protein (LAG-3), and mucin domain molecule 3 (TIM-3). (19) The high levels of ICIs induce T cell inhibition and represent one of the major mechanisms of HCC immune escape. Thus, immune checkpoint inhibitors (ICIs) have rapidly progressed in the treatment of HCC, but monotherapy with programmed cell death protein-1 (PD-1) antibody only caused tumor regression in 20% of the patients. (20, 21) However, PD-1 combined with vascular endothelial growth factor (VEGF) inhibitors may improve the immune response of the tumor microenvironment. This combination could increase the infiltration of CD8+ T cells in the TME by temporarily normalizing the tumor vessels, an effect of blocking VEGF, and amplifying the value of PD-1 antibody. (22) In the IMbrave 150 research, the combination of PD-L1 antibody and VEGF inhibitor demonstrated a prolonged survival and higher response rate compared with sorafenib, causing a 42% risk reduction in mortality; thus, it is recommended as the first-line treatment for unresectable HCC. (23)

TACE, as an local-regional therapy (LRT), may induce “immunogenic cell death” by releasing tumor antigens and eliciting damage-associated molecular patterns, to facilitate antitumor immunity. (24, 25) In addition, TACE could cause an increase in VEGF and PD-L1 expression because of the hypoxic microenvironment after embolization. (26, 27) The molecularly targeted anti-cancer agents combined with the PD-1 antibody would be a promising complement to TACE. However, whether patients with uHCC can obtain core survival benefits from TACE combined with ICIs and molecularly targeted drugs remains unclear, and few studies have focused on this issue.

Lenvatinib is a novel tyrosine kinase inhibitor (TKI), which was approved in 2018 as the first-line treatment of uHCC, proven to be non-inferior to sorafenib in increasing the overall survival (OS) in patients with HCC in clinical trials. (28) Camrelizumab (AiRuiKa™) is a humanized high-affinity IgG4-kappa PD-1 monoclonal antibody being developed by Jiangsu Hengrui Medicine Co. Ltd (Jiangsu Hengrui Medicine, Jiangsu, China) for the treatment of various malignancies including HCC, exhibiting promising antitumor activity and an acceptable safety profile. (29, 30)

In this study, we assessed the efficacy and safety of lenvatinib combined with camrelizumab plus TACE (TACE-LEN-C) in patients with uHCC in two centers in China and compared them with those of TACE combined with lenvatinib (TACE-LEN).

## Patients and methods

### Patients and study design

Patients who received TACE-LEN (n=52) or TACE-LEN-C (n=31) between September 2018 and May 2021 were enrolled in this retrospective observational study. All patients were pathologically or clinically diagnosed with HCC according to the guidelines of the American Association for the Study of Liver Diseases (31). All patients were confirmed unresectable by a multi-disciplinary team and all patients did not receive systematic anti-cancer therapy before the combination treatments.

The inclusion criteria were as follows: unresectable HCC treated with TACE-LEN-C or TACE-LEN; age between 18 and 75 years, and the presence of at least one target lesion with measurable diameter and arterial enhancement according to the modified Response Evaluation Criteria in Solid Tumors (mRECIST). (32) The exclusion criteria were as follows: presence of serious complications, such as severe dysfunction of the kidney, lung, or heart; having undergone other treatments during this period, such as thermal ablation, external beam radiotherapy, or percutaneous ethanol injection; the presence of other malignant tumors in addition to HCC; and incomplete data.

This study was approved by the ethics committee of the Wuhan Union Hospital, Huazhong University of Science and Technology and The Fifth Medical Center of Chinese, PLA General Hospital.

### Treatment

TACE was performed by puncturing the right femoral artery. A 5-F catheter and 3-F microcatheter were used to identify the tumor-supplying artery. Lipiodol and doxorubicin hydrochloride were mixed and injected into the tumor-supplying artery *via* a microcatheter. Then, an appropriate amount of gelatin sponge was injected to seal the tumor’s blood supplying artery.

In the TACE-LEN-C group, patients received camrelizumab and lenvatinib in one week after TACE. Patients received lenvatinib orally once daily at a dose of 8 mg (body weight <60 kg) or 12 mg (body weight ≥60 kg) and 200 mg camrelizumab intravenously once every 3 weeks until no clinical benefits be observed or unacceptable toxicity. (23) The mean time for patients receiving lenvatinib was 15.5 months (range 3-23 months) and 14.3 months for patients with camrelizumab (range 3.5-22 months). Patients receiving another TACE is according to the CT or MRI imaging evaluation based on the mRECIST criteria. The patients were recommended to receive another TACE if there were residual tumors (partial response, PR or stable disease, SD). However, patients were not recommended to receive TACE if the tumor continuously progressed after two TACEs because these patients were considered as TACE resistance. (17) For these patients, the lenvinib or camrelizumab should be taken if the investigators observed evidence of clinical benefits were absent. Lenvatinib and camrelizumab were discontinued for 3 days before and after TACE. In the TACE-LEN group, patients received lenvatinib, as in the TACE-LEN-C group.

### Efficacy and safety

Adverse events (AEs) were monitored and recorded by experienced nurses who were blinded to the treatment, according to the National Cancer Institute Common Terminology Criteria for Adverse Events version 5.0.

Treatment responses were assessed based on contrast-enhanced abdominal CT or MR imaging according to mRECIST. Complete response (CR) was defined as all target lesions disappeared, no new lesions appeared, and tumor markers were normal for at least 4 weeks. Partial response (PR) was defined as the reduction of the sum of the largest diameters of target lesions ≥30% and maintained for at least 4 weeks. Stable disease was defined as the sum of the largest diameters of target lesions did not shrink to the standard of PR, or enlarged to the standard of progressive disease (PD). PD was defined as the sum of the largest diameters of target lesions increased by at least ≥20%, or new lesions appear. The objective response rate (ORR) was defined as the incidence of complete response and partial response. The disease control rate (DCR) was defined as the incidence of complete response (CR), partial response (PR), and stable disease (SD). Progression-free survival (PFS) was defined as the time from the initiation of treatment to first tumor progression (first PD), death or the last follow-up in censored data. OS) was defined as the time from the initiation of treatment until death or the last follow-up in censored data.

### Follow-up

The interval between the follow-ups was 4–6 weeks. During the follow-up, the patients underwent laboratory examinations, physical examinations and a thorough inquiry to record the AEs. Laboratory examinations included AFP, ALT, AST, and others. Contrast-enhanced CT or MRI was performed to identify the intrahepatic recurrent or residual tumors. Once an intrahepatic viable tumor appeared, another TACE was performed according to the consensus of the patient and their attending physician.

### Statistical analysis

To compare the differences in baseline characteristics between the two groups of patients, Fisher’s exact test or χ2 test was used for categorical variables, presented as numbers (percentages), and Student’s t-test were used for continuous variables, presented as mean ± standard deviation. Kaplan–Meier analysis was used to plot the OS and PFS curves, and significance was calculated using the log-rank test. Cox proportional regression analysis was used to calculate potential factors that might influence the survival of all patients. Factors with p-values no more than 0.1 in the univariable analysis were included in the multivariate analysis. Differences were considered statistically significant when the bilateral p-value was ≤ 0.05. All statistical analyses were conducted using SPSS 24.0 and R.

## Results

### Baseline statistics

**Figure 1:**
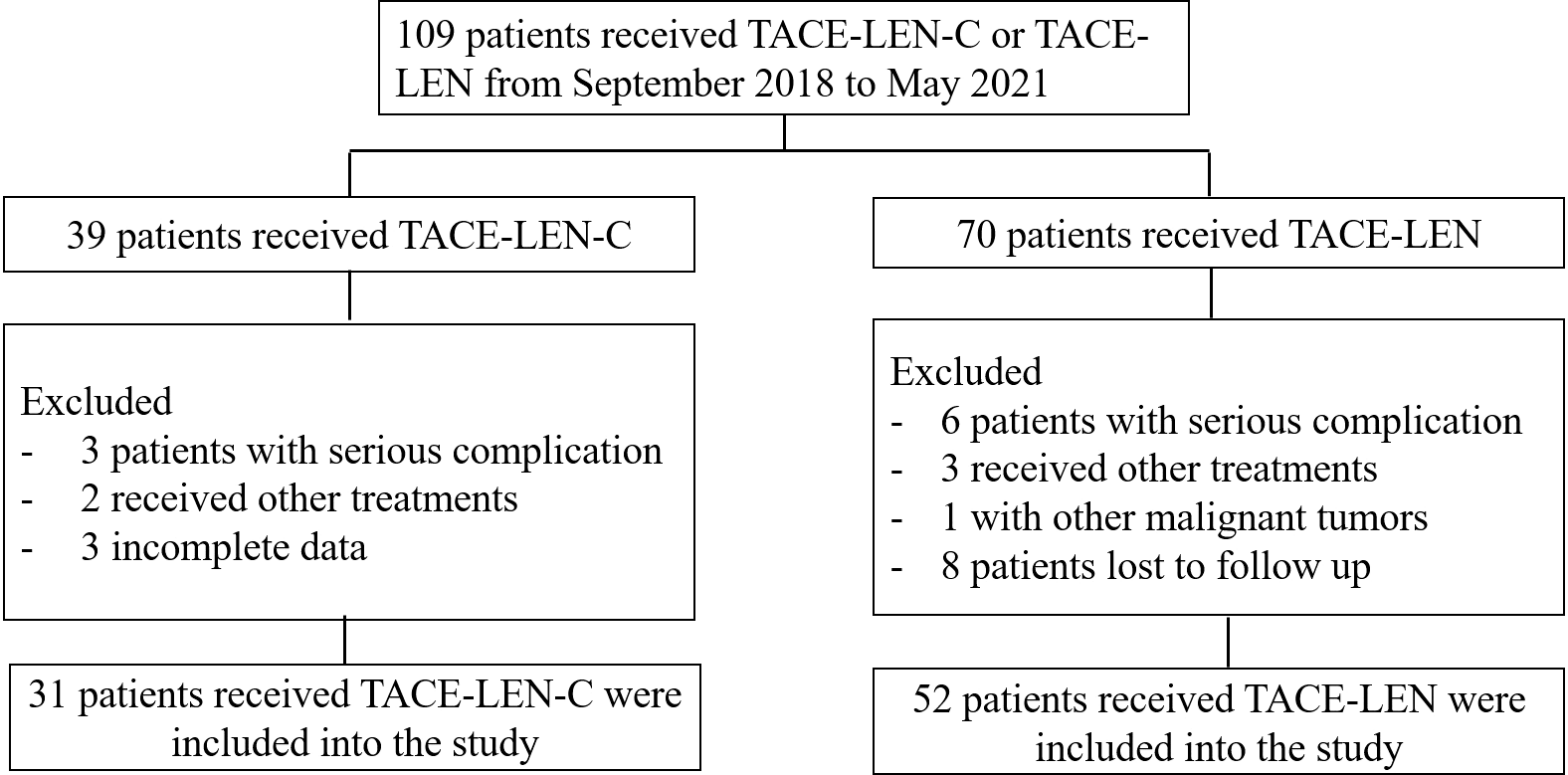
Flow chart illustrating the selection of patients; TACE-LEN-C, transcatheter arterial chemoembolization with lenvatinib plus camrelizumab; TACE-LEN, transcatheter arterial chemoembolization with LEN.

During the follow-up, there were a total of 109 patients (61 in Wuhan Union hospital, 48 in The Fifth Medical Center of Chinese, PLA General Hospital) with uHCC who received TACE-LEN-C (n=39) or TACE - LEN (n=70). However, 8 and 18 patients in the TACE-LEN-C and TACE-LEN groups, respectively were excluded according to the exclusion criteria. The flowchart is shown in **Figure 1**. Finally, 31 patients in the TACE-LEN-C group and 52 in the TACE-LEN group were enrolled in the study. There was no statistical difference in baseline variables between the two groups (**Table 1**).

**Table 1:** Baseline characteristics of patients between the two groups.

Characteristics	TACE-LEN (N=52)	TACE-LEN-C (N=31)	*P* value
**Age(years)**	51.77 ± 9.791	54.84 ± 9.249	0.332
**Genders**			0.511
Male	46(88.5%)	25(80.6%)	
Female	6(11.5%)	6(19.4%)	
**ECOG performance**			0.115
0	22(42.3%)	19(61.3%)	
1	30(57.7%)	12(38.7%)	
**BCLC stage**			0.814
B	17(32.7%)	11(35.5%)	
C	35(67.3%)	20(64.5%)	
**Extrahepatic metastases**			0.548
Yes	32 (61.5%)	17 (54.8%)	
No	20 (38.5%)	14 (45.2%)	
**HBV infection**			0.389
Yes	44(84.6%)	29(93.5%)	
No	8(15.4%)	2(6.5%)	
**AFP (ng/ml)**			1.000
>400	21(40.4%)	12(38.7%)	
≤400	31(59.6%)	19(61.3%)	
**Child-Pugh Class**			0.576
A	43(82.7%)	24(77.4%)	
B	9(17.3)	7(22.6%)	
**Tumors number**			0.232
≤3	49(94.2%)	27(87.1%)	
>3	3(5.8%)	4(12.9%)	
**ALT(IU/L)**	37.90 ± 21.00	36.42 ± 24.67	0.443
**AST(IU/L)**	49.19 ± 26.44	42.03 ± 20.73	0.241
**TB (µmol/L)**	16.66 ± 6.60	16.83 ± 5.85	0.696
**PLR**	132.33 ± 70.49	119.13 ± 70.84	0.207
**NLR**	3.18 ± 2.38	2.80 ± 2.22	0.592
**Albumin(g/dl)**	37.70 ± 4.46	35.65 ± 4.85	0.085
**PT(S)**	13.46 ± 1.62	13.61 ± 2.97	0.226
**Tumor size(cm)**	7.65 ± 4.86	8.31 ± 4.80	0.392
**TACE Sessions**	4.38 ± 2.39	3.68 ± 2.01	0.951

ECOG, Eastern Cooperative Oncology Group; BCLC Barcelona Clinic Liver Cancer; HBV, hepatitis B virus; AFP, a-fetoprotein; TACE, transarterial chemoembolization; ALT, alanine aminotransferase; AST, aspartate aminotransferase; TB, total bilirubin; PLR, platelet-to-lymphocyte; NLR, neutrophil-to-lymphocyte ratio; PT, prothrombin time; TACE-LEN, transcatheter arterial chemoembolization with Lenvatinib; TACE-LEN-C, transcatheter arterial chemoembolization with Lenvatinib plus camrelizumab.

### Tumor response and patient survival

Tumor responses of the two groups were shown in **Table 2**. There were 3, 19, 7, 2 patients in the TACE-LEN-C group had CR, PR, SD, and progressive disease (PD), respectively, both PD patients were confirmed as immune-related confirmed progressed disease(iCPD) by immune-related response criteria in solid tumors (iRECIST) (33). There were 1, 21, 20, and 10 patients in the TACE-LEN group with CR, PR, SD, and PD. The patients in the TACE-LEN-C group had a better ORR than those in the TACE-LEN group (71% vs. 42.3% by the mRECIST, *P*=0.023). The DCR in the two groups demonstrated no statistical difference (93.5% in TACE-LEN-C, 80.8% in TACE-LEN, *P*= 0.195).

**Table 2:** Tumor response in both groups.

Tumor response	TACE-LEN (N=52)	TACE-LEN-C (N=31)	*P* value
**CR**	1(1.9%)	3(9.7%)	
**PR**	21(40.3%)	19(61.3%)	
**SD**	20(38.5%)	7(22.6%)	
**PD**	10(19.2%)	2(6.5%)	
**ORR**	22(42.3%)	22(71.0%)	0.023
**DCR**	42(80.8%)	29(93.5%)	0.195

Data are presented as n (%), CR, complete response; PR, partial response; SD, stable disease; PD, progressive disease; ORR, objective response rate; DCR, disease control rate; TACE-LEN, transcatheter arterial chemoembolization with Lenvatinib; TACE-LEN-C, transcatheter arterial chemoembolization with Lenvatinib plus camrelizumab.

**Figure 2:**
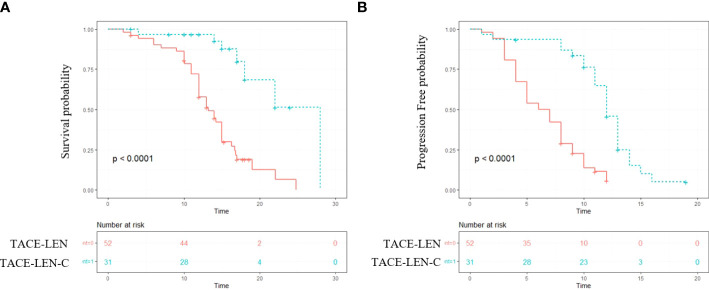
The Kaplan–Meier (KM) curves for patients with unresectable hepatocellular carcinoma who received the treatment of TACE-LEN-C or TACE-LEN (TACE-LEN-C, transcatheter arterial chemoembolization with lenvatinib plus camrelizumab; TACE-LEN, transcatheter arterial chemoembolization with LEN): **(A)** the KM curves of overall survival time; **(B)** the KM curves of profession-free time.

The median follow-up period of all the patients was 14.2 months (range 7.2–25.2 months). During the follow-up, 40 (76.9%) and 11(35.5%) patients died in the TACE-LEN and TACE-LEN-C groups, respectively. The median OS was significantly longer in the TACE-LEN-C group than that in the TACE-LEN group (18.9 vs. 13.9 months, *P*<0.001) (**Figure 2A**). The 6 and 12 months survival rates in the TACE-LEN-C and TACE-LEN-C groups were 96.7%, 95.7% and 88.2%, and 55.1%, respectively (**Table 3**). The median PFS of the TACE-LEN-C and TACE-LEN groups was 12.5 vs 6.6 months, respectively (**Figure 2B**). The 6 and 12 months progression-free rates of the TACE-LEN-C and TACE-LEN groups were 93.3%, 42.3% and 50%, 0%, respectively (**Table 3**).

**Table 3:** Rate of overall survival and progression free survival at 6 months and 12 months.

Rate, %	TACE-LEN (N=52)	TACE-LEN-C (N=31)
**OS 6 months**	88.2	96.7
**OS 12 months**	55.1	95.7
**PFS 6 months**	50	93.3
**PFS 12 months**	0	42.3

OS, overall survival; PFS, progression-free survival; TACE-LEN, transcatheter arterial chemoembolization with Lenvatinib; TACE-LEN-C, transcatheter arterial chemoembolization with Lenvatinib plus camrelizumab.

### Predictive factors affecting PFS and OS

In the univariate analysis, the number of tumors (hazards ratio [HR]: 3.192; 95% confidence interval [CI]: 1.570–6.492; *P*=0.001), BCLC stage (HR: 1.567; 95% CI: 0.939–2.615; *P*=0.085), and treatment (HR: 0.351; 95% CI: 0.211–0.584; *P*< 0.01) were the potential factors affecting PFS, and the potential factors associated with OS included age (HR: 0.967; 95% CI: 0.952–1.001; *P*=0.060), sex (HR: 0.366; 95% CI: 0.112–1.195; *P*=0.096), ECOG performance (HR: 2.926; 95% CI: 1.578–5.427; *P*=0.001), number of tumors (HR: 4.783; 95% CI: 1.476–15.513; *P*=0.009), BCLC stage (HR: 1.911; 95% CI: 1.006–3.630; *P*=0.048), Extrahepatic metastases (HR: 0.578; 95% CI: 0.325~1.453; P=0.12) and treatment (HR: 0.171; 95% CI: 0.072–0.403; *P*<0.001). In the multivariate analysis, two independent factors affecting PFS were identified: number of tumors (HR: 2.212; 95% CI: 1.022–4.790; *P*=0.044) and treatment (HR: 0.451; 95% CI: 0.259–0.784; *P*=0.005). Three independent factors affected OS: number of tumors (HR: 2.250; 95% CI: 1.034–4.894; *P*=0.041), BCLC stage (HR: 1.738; 95% CI: 1.025–2.947; *P*=0.040) and treatment (HR: 0.381; 95% CI: 0.201–0.725; *P*=0.003). (**Tables 4**, **5**)

**Table 4:** Univariate and multivariate analysis of prognostic factors for PFS.

Variable	Univariate analysis		Multivariate analysis	
	HR (95%CI)	*P* value	HR (95%CI)	*P* value
**Age**	0.992(0.972~1.013)	0.449		
**Gender**		0.158		
Male	Reference			
Female	0.587(0.280~1.229)			
**ECOG performance**		0.250		
0	Reference			
1	1.319(0.823~2.114)			
**Number of tumors**		**0.001**		**0.044**
>3	Reference		Reference	
≤3	3.192(1.570~6.492)		2.212(1.022~4.790)	
**HBV infection**		0.463		
No	Reference			
Yes	0.758(0.361~1.589)			
**Child-Pugh class**		0.210		
A	Reference			
B	0.651(0.333~1.274)			
**BCLC stage**		**0.085**		0.056
B	Reference		Reference	
C	1.567(0.939~2.615)		1.662(0.987~2.797)	
**Extrahepatic metastases**		0.153		
Yes	Reference			
No	0.845 (0.563~1.351)			
**AFP (ng/ml)**		0.806		
>400	Reference			
≤400	1.063(0.655~1.725)			
**Tumor size (cm)**	1.005(0.952~1.060)	0.862		
**PLR**	1.000(0.996~1.003)	0.929		
**NLR**	0.904(0.794~1.028)	0.124		
**ALT(IU/L)**	1.001(0.997~1.005)	0.512		
**AST (IU/L)**	1.001(0.992~1.009)	0.892		
**Albumin (g/dL)**	1.040(0.987~1.096)	0.144		
**TB (µmol/L)**	1.019(0.978~1.062)	0.370		
**PT(s)**	1.042(0.956~1.135)	0.348		
**TACE Sessions**	0.971(0.896~1.052)	0.468		
**Treatment**		**<0.001**		**0.005**
TACE-LEN	Reference		Reference	
TACE-LEN-C	0.351(0.211~0.584)		0.451(0.259~0.784)	

HR, hazard ratio; CI, confidence interval; BCLC, Barcelona Clinic Liver Cancer; HBV, hepatitis B; AFP, alpha-fetoprotein; ALT, alanine transaminase; AST, aspartate aminotransferase; HCC, hepatocellular carcinoma; PFS, progression-free survival; TACE-LEN, transcatheter arterial chemoembolization with Lenvatinib; TACE-LEN-C, transcatheter arterial chemoembolization with Lenvatinib plus camrelizumab. Bold values signifies important information in the Statistical analysis section.

**Table 5:** Univariate and multivariate analysis of prognostic factors for OS.

Variable	Univariate analysis		Multivariate analysis	
	HR (95%CI)	*P* value	HR (95%CI)	*P* value
**Age**	0.976(0.952~1.001)	**0.060**	1.004(0.980~1.028)	0.753
**Gender**		**0.096**		0.168
Male	Reference		Reference	
Female	0.366(0.112~1.195)		0.589(0.277~1.250)	
**ECOG performance**		**0.001**		0.276
0	Reference		Reference	
1	2.926(1.578~5.427)		0.735(0.422~1.279	
**Number of tumors**		**0.009**		**0.041**
>3	Reference		Reference	
≤3	4.785(1.476~15.513)		2.250(1.034~4.894)	
**HBV infection**		0.345		
No	Reference			
Yes	0.654(0.271~1.577)			
**Child-Pugh class**		0.299		
A	Reference			
B	0.651(0.290~1.463)			
**BCLC stage**		**0.048**		**0.040**
B	Reference		Reference	
C	1.911(1.006~3.630)		1.738(1.025~2.947)	
				
**Extrahepatic metastases**		0.120		
Yes	Reference			
No	0.578(0.325~1.453)			
**AFP (ng/ml)**		0.734		
>400	Reference			
≤400	0.901(0.494~1.643)			
**Tumor size (cm)**	1.007(0.943~1.076)	0.827		
**PLR**	1.001(0.996~1.006)	0.677		
**NLR**	1.002(0.895~1.166)	0.750		
**ALT(IU/L)**	1.007(0.996~1.019)	0.216		
**AST (IU/L)**	1.006(0.996~1.016)	0.243		
**Albumin (g/dL)**	1.037(0.970~1.109)	0.290		
**TB (µmol/L)**	1.026(0.977~1.077)	0.303		
**PT(s)**	1.018(0.897~1.156)	0.783		
**TACE Sessions**	1.035(0.933~1.148)	0.514		
**Treatment**		**<0.001**		**0.003**
TACE-LEN	Reference		Reference	
TACE-LEN-C	0.171(0.072~0.403)		0.382(0.201~0.725)	

HR, hazard ratio; CI, confidence interval; BCLC, Barcelona Clinic Liver Cancer; HBV, hepatitis B; AFP, alpha-fetoprotein; ALT, alanine transaminase; AST, aspartate aminotransferase; HCC, hepatocellular carcinoma; OS, overall survival; TACE-LEN, transcatheter arterial chemoembolization with Lenvatinib; TACE-LEN-C, transcatheter arterial chemoembolization with Lenvatinib plus camrelizumab. Bold values signifies important information in the Statistical analysis section.

### Safety

The most common TACE-related AEs were post-embolization syndrome that induced fever (80.8% vs. 71%), pain (61.5% vs. 67.8), nausea (65.3% vs. 61.3%), and vomiting (32.6% vs. 35.4%) in the TACE-LEN and TACE-LEN-C groups. Grade 3 or 4 AEs with an incidence of more than 5% included fever in both groups and nausea in the TACE-LEN group. Drug-related AEs demonstrated a similar incidence in both groups, with no grade 3 or 4 AEs of > 5% (only two cases of hypertension in the TACE-LEN group and one case of hypertension in the TACE-LEN-C group). Drug-related AEs included elevated ALT levels (23.1% vs. 25.8%), insomnia (3.8% vs. 3.2%), proteinuria (17.3% vs. 19.4%), ventosity (13.5% vs. 11.8%), hypertension (25% vs. 25.8), hypothyroidism (0% vs. 12.9%) and hand-foot skin reaction (21.2% vs. 22.6%). All AEs are listed in **Table 6**.

**Table 6:** The adverse events of patients after receiving TACE-LEN or TACE-LEN-C.

Adverse event	TACE-LEN(n=52)		TACE-LEN-C(n=31)	
	Any grade (n, %)	Grade 3 or 4 (n, %)	Any grade (n, %)	Grade 3 or 4 (n, %)
**Fever**	42(80.8)	5(9.6)	22(71.0)	3(9.7)
**Pain**	32(61.5)	2(3.8)	21(67.8)	1(3.2)
**Nausea**	34(65.3)	3(5.8)	19(61.3)	1(3.2)
**Vomiting**	17(32.6)	3(3.8)	11(35.4)	1(3.2)
**Elevated ALT**	12 (23.1)	1 (1.9)	8 (25.8)	0 (0)
**Insomnia**	2 (3.8)	0 (0)	1 (3.2)	0 (0)
**Proteinuria**	9 (17.3)	0 (0)	6 (19.4)	0 (0)
**Ventosity**	7 (13.5)	0 (0)	4 (11.8)	0 (0)
**Hypertension**	13(25.0)	2(3.8)	8(25.8)	1(3.2)
**Hypothyroidism**	0(0)	0(0)	4(12.9)	0(0)
**Hand-foot skin reaction**	11(21.2)	0(0)	7(22.6)	0(0)

## Discussion

In the past few years, many studies have been conducted to identify an appropriate systematic therapy protocol for patients with uHCC treated with TACE. The TACTICS trial with sorafenib and TACE in patients with uHCC indicated that TACE combined with antineoplastic agents is an independent predictor of prognosis for uHCC. (17) Several phase I or II trials have been conducted to evaluate the safety and efficacy of TACE plus ICIs (NCT03143270, NCT03572582, NCT03397654). In addition, there are some phase III RCTs on the combination of TKI and ICIs plus TACE, such as the LEAP-012 trial (NCT04246177) using lenvatinib plus pembrolizumab vs. placebo in combination with TACE, CheckMate 74W trial (NCT04340193) using nivolumab and ipilimumab plus TACE, and EMERALD study (NCT03778957) using durvalumab and bevacizumab plus TACE. However, these results remain unclear. Thus, we have summarized and described our experience of using lenvatinib and camrelizumab plus TACE in our centers.

Our results revealed that uHCC patients who received TACE combined with lenvatinib plus camrelizumab had prolonged OS and PFS compared with those who received TACE combined with lenvatinib. In the multivariate analyses, combination with camrelizumab was an independent predictor for better OS and PFS. The combination also demonstrated a higher ORR (71% vs. 42.3%, *P*=0.023) in patients who received TACE-LEN-C than in those who received TACE-LEN. Interestingly, in a previous study, the ORR of lenvatinib plus pembrolizumab was 46%, lower than that of the TACE-LEN-C group. (34) Thus, this combination may result in an obvious improvement in controlling the tumor locally, which may result in an increase in patient’s survival. In previous studies, the combination of TACE-LEN-C also demonstrated a significant increase in tumor responses and survival benefits for uHCC patients. (35–37) Although, in this study, there was no statistically significant difference in DCR (93.5% in TACE-LEN-C, 80.8% in TACE-LEN, *P*=0.195), the combination still deserves consideration as a prioritized treatment strategy for uHCC patients.

These encouraging effects may be attributed to a stronger local and systematic immune response. A recent study demonstrated that the failure of anti-PD-1 partly results from the imbalance between CD8+ T cells and tumor burden, and the therapeutic efficacy of anti-PD-1 is positively associated with the ratio of CD8+ T cell invigoration to the tumor burden (measured as the sum of the long axis of all lesions, cm). (38) TACE can reduce tumor burden and elicit a response against cell death antigens, causing immunogenic cell death(ICD). (27) In addition, emerging evidence suggests that the ectopic overexpression of VEGF results in a highly abnormal vasculature, preventing the infiltration of immune effector cells (especially CD8+T cells). (39, 40) Thus, TACE-LEN-C could reduce tumor burden, increase the infiltration of CD8+T cells, and alleviate the inhibitory effect of CD8+T cells, leading to local and systemic immune activation. Further studies are required to verify this hypothesis.

Tumor burden has been proven to lead to poor prognosis in patients with uHCC who received TACE or immunotherapy. (41, 42) In our study, the Cox model was used to reduce potential factors that might influence the outcomes. Patients with ≤ 3 tumors could increase the all-cause mortality risk and tumor progression risk compared to patients with > 3 tumors. There were 3 patients (5.8%) in the TACE-LEN group and 4 patients (12.9%) in the TACE-LEN group with tumor number > 3. The number of patients was small which might lead to statistical bias. Thus, wo hope the future studies conducted by us or other studies can include more patients with tumor number > 3 to confirm the results generated by the current study. Patients with BCLC stage C could increase all-cause mortality risk compared to patients with BCLC stage B. However, after excluding other factors that might influence the outcomes, TACE-LEN-C could reduce tumor progression risk and the all-cause mortality risk compared to TACE-LEN, which might indicate that patients with uHCC could get more survival benefits from TACE-LEN-C than TACE-LEN.

In terms of AEs, our study suggested that TACE-LEN-C was well-tolerated and led to manageable side effects in patients with uHCC. The most commonly reported drug-related toxicities were elevated ALT, insomnia, proteinuria, ventosity, hypertension, hypothyroidism and hand-foot skin reaction, similar to previous studies. (35, 36) Furthermore, TACE-LEN-C did not increase TACE-related complications in patients with uHCC, specifically post-embolization syndrome. No permanent adverse sequelae or treatment-related deaths were reported. Thus, these results suggest that TACE-LEN-C was well-tolerated by patients with uHCC.

Our study had some limitations. This was a retrospective study with a small sample size, and further prospective studies are needed to confirm the efficacy of TACE-LEN-C. In addition, a recent study indicated that lenvatinib is better than sorafenib in patients with hepatitis B virus (HBV), (43) and most patients in our study had HBV. Thus, more studies need to be conducted to confirm the efficacy in other types of patients and more types of TKI +PD-1 antibody combinations need to be tested to determine the best combination as a supplementary systematic therapy to TACE.

## Conclusion

Our study showed that patients who received TACE-LEN-C demonstrated a better tumor response and survival benefits with tolerable AEs. TACE-LEN-C is a safe and effective treatment for patients with uHCC and deserves consideration as a prioritized option.

## Data availability statement

The raw data supporting the conclusions of this article will be made available by the authors, without undue reservation.

## Ethics statement

The studies involving human participants were reviewed and approved by Wuhan Union Hospital, Huazhong University of Science and Technology and The Fifth Medical Center of Chinese, PLA General Hospital. The ethics committee waived the requirement of written informed consent for participation.

## Author contributions

Conception and design: CZ, FX, and LC; administrative support: CZ; provision of the study materials or patients: CZ and FX; collection and assembly of data: BS, LCZ, TS, YR, YC, YXG, and YSG; data analysis and interpretation: BS, TS, and LJZ; Manuscript writing: All authors. Final approval of manuscript: All authors.

## Funding

This study was supported by the National Natural Science Foundation of China (No. 81873919).

## Conflict of interest

The authors declare that the research was conducted in the absence of any commercial or financial relationships that could be construed as a potential conflict of interest.

## Publisher’s note

All claims expressed in this article are solely those of the authors and do not necessarily represent those of their affiliated organizations, or those of the publisher, the editors and the reviewers. Any product that may be evaluated in this article, or claim that may be made by its manufacturer, is not guaranteed or endorsed by the publisher.
